# Emergency Myelopoiesis Distinguishes Multisystem Inflammatory Syndrome in Children From Pediatric Severe Coronavirus Disease 2019

**DOI:** 10.1093/infdis/jiae032

**Published:** 2024-01-31

**Authors:** Katerina Roznik, Temesgen E Andargie, T Scott Johnston, Oren Gordon, Yi Wang, Nadine Peart Akindele, Deborah Persaud, Annukka A R Antar, Yukari C Manabe, Weiqiang Zhou, Hongkai Ji, Sean Agbor-Enoh, Andrew H Karaba, Elizabeth A Thompson, Andrea L Cox

**Affiliations:** W. Harry Feinstone Department of Molecular Microbiology and Immunology, Johns Hopkins Bloomberg School of Public Health; Division of Infectious Diseases, Department of Medicine, Johns Hopkins University School of Medicine, Baltimore; Genomic Research Alliance for Transplantation and Laboratory of Applied Precision Omics, National Heart, Lung, and Blood Institute, National Institutes of Health, Bethesda, Maryland; Department of Biology, Howard University, Washington, District of Columbia; Division of Infectious Diseases, Department of Medicine, Johns Hopkins University School of Medicine, Baltimore; Infectious Diseases Unit, Department of Pediatrics, Faculty of Medicine, Hadassah Medical Center, Hebrew University of Jerusalem, Israel; Department of Pediatrics, Johns Hopkins University School of Medicine; Department of Biostatistics, Johns Hopkins Bloomberg School of Public Health, Baltimore; Department of Pediatrics, Johns Hopkins University School of Medicine; Center for Biologics Evaluation and Research, United States Food and Drug Administration, Silver Spring, Maryland; W. Harry Feinstone Department of Molecular Microbiology and Immunology, Johns Hopkins Bloomberg School of Public Health; Department of Pediatrics, Johns Hopkins University School of Medicine; Division of Infectious Diseases, Department of Medicine, Johns Hopkins University School of Medicine, Baltimore; Division of Infectious Diseases, Department of Medicine, Johns Hopkins University School of Medicine, Baltimore; Department of Biostatistics, Johns Hopkins Bloomberg School of Public Health, Baltimore; Department of Biostatistics, Johns Hopkins Bloomberg School of Public Health, Baltimore; Division of Infectious Diseases, Department of Medicine, Johns Hopkins University School of Medicine, Baltimore; Genomic Research Alliance for Transplantation and Laboratory of Applied Precision Omics, National Heart, Lung, and Blood Institute, National Institutes of Health, Bethesda, Maryland; Division of Infectious Diseases, Department of Medicine, Johns Hopkins University School of Medicine, Baltimore; Division of Infectious Diseases, Department of Medicine, Johns Hopkins University School of Medicine, Baltimore; W. Harry Feinstone Department of Molecular Microbiology and Immunology, Johns Hopkins Bloomberg School of Public Health; Division of Infectious Diseases, Department of Medicine, Johns Hopkins University School of Medicine, Baltimore

**Keywords:** MIS-C, pediatric COVID-19, emergency myelopoiesis, cell-free DNA, IL-27

## Abstract

**Background:**

Multisystem inflammatory syndrome in children (MIS-C) is a hyperinflammatory condition caused by recent infection with severe acute respiratory syndrome coronavirus 2, but the underlying immunological mechanisms driving this distinct syndrome are unknown.

**Methods:**

We utilized high-dimensional flow cytometry, cell-free (cf) DNA, and cytokine and chemokine profiling to identify mechanisms of critical illness distinguishing MIS-C from severe acute coronavirus disease 2019 (SAC).

**Results:**

Compared to SAC, MIS-C patients demonstrated profound innate immune cell death and features of emergency myelopoiesis (EM), an understudied phenomenon observed in severe inflammation. EM signatures were characterized by fewer mature myeloid cells in the periphery and decreased expression of HLA-DR and CD86 on antigen-presenting cells. Interleukin 27 (IL-27), a cytokine known to drive hematopoietic stem cells toward EM, was increased in MIS-C, and correlated with immature cell signatures in MIS-C. Upon recovery, EM signatures decreased and IL-27 plasma levels returned to normal levels. Despite profound lymphopenia, we report a lack of cfDNA released by adaptive immune cells and increased CCR7 expression on T cells indicative of egress out of peripheral blood.

**Conclusions:**

Immune cell signatures of EM combined with elevated innate immune cell-derived cfDNA levels distinguish MIS-C from SAC in children and provide mechanistic insight into dysregulated immunity contributing toward MIS-C, offering potential diagnostic and therapeutic targets.

## Graphical Abstract

**Graphical Abstract:**
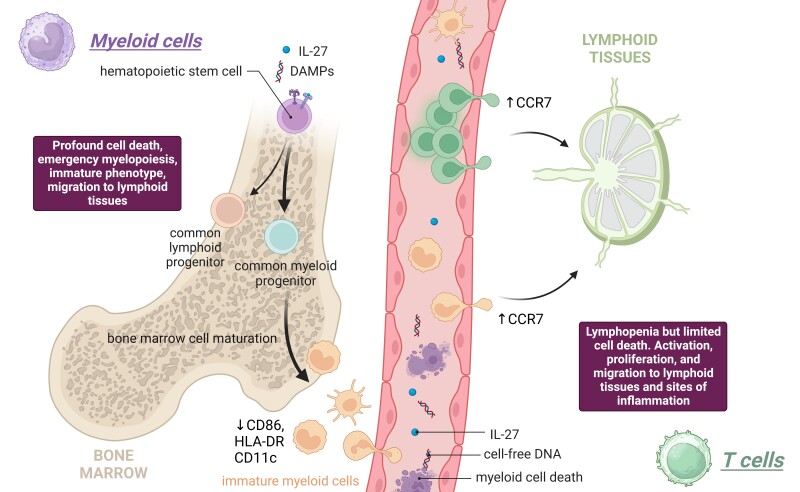

## Teaser

This article explores underlying mechanisms of the multisystem inflammatory syndrome in children (MIS-C) and provides novel insights into T-cell and myeloid cell fates contributing to MIS-C pathology.

Multisystem inflammatory syndrome in children (MIS-C) affects children 2–6 weeks after infection with severe acute respiratory syndrome coronavirus 2 (SARS-CoV-2) and represents a late abnormal immune response to viral infection. Clinically, MIS-C presents with multiorgan dysfunction and has been characterized by blood cell dysregulation, including lymphopenia, neutrophilia, and elevated inflammatory biomarkers and cytokines [1–9]. It was reported recently that the syndrome is much more common and severe than previously thought, with 17 cases per 100 pediatric hospitalizations related to SARS-CoV-2 infection [10]. Additionally, MIS-C causes cardiac complications in >80% of patients and has a mortality rate of 1%–6% depending on the number of organs affected [10, 11].

Because knowledge of this syndrome is still emerging, it can be challenging to rapidly distinguish MIS-C from severe acute coronavirus disease 2019 (hereafter “SAC”) in hospitalized children. Moreover, the causes of immune dysregulation in MIS-C and why some children progress to MIS-C after recovering from coronavirus disease 2019 (COVID-19) remain unknown. In this study, we examined immunologic differences between children presenting in the beginning of the pandemic with MIS-C, SAC, or fully recovered from mild COVID-19 (CFR) using flow cytometry, plasma cytokine and chemokine profiling, and analysis of cell-free DNA (cfDNA). We also utilized a publicly available single-cell RNA sequencing (scRNAseq) dataset comparing patients hospitalized with MIS-C and pediatric healthy controls (HCs) to verify our findings [12]. We focused on myeloid cells and T cells and provide evidence that MIS-C pathology is associated with significant innate immune dysregulation, emergency myelopoiesis (EM) signature, and innate cell death, features generally absent in children hospitalized with SAC. We report reduced expression of costimulatory molecules on antigen-presenting cells (APCs) in MIS-C associated previously with the phenomenon of EM and reported in the acute phase of severe COVID-19 in hospitalized adults [13–15]. EM is defined as inflammation-induced hematopoiesis to replenish myeloid cells in the periphery and is critical to infection control [16, 17]. Quiescent hematopoietic stem cells (HSCs) can be activated to proliferate and preferentially differentiate to myeloid cells by damage-associated molecular patterns (DAMPs), pathogen-associated molecular patterns, and cytokines such as interferon-γ, interleukin (IL) 3, IL-11, granulocyte colony-stimulating factor (G-CSF), and thrombopoietin (TPO) [18]. However, recent studies identify the cytokine IL-27 as one of the strongest inducers of continuous HSC expansion, which was significantly elevated in MIS-C participants [17–21]. Detecting EM signatures in children experiencing febrile illness could significantly impact the diagnosis of MIS-C, which lacks precise laboratory diagnostic tools, and early detection could pave the way for potential future immunomodulatory therapies.

## METHODS

### Study Design and Recruitment

**Figure 1.**
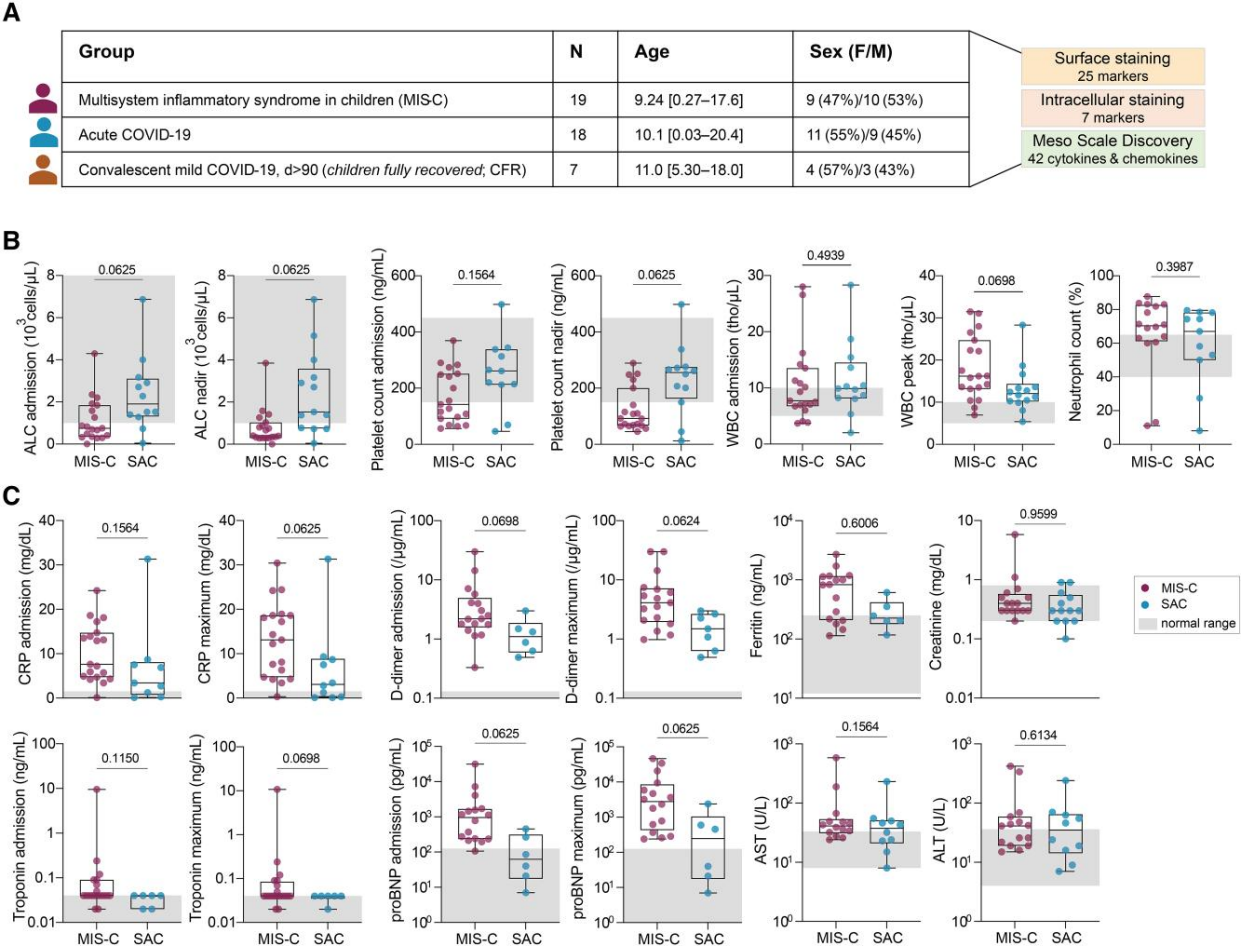
Study cohort and inflammatory laboratory values. *A*, Basic demographic characteristics of the study cohort. *B* and *C*, Inflammatory laboratory values. Normal ranges are depicted in gray shading. Significance was tested using Wilcoxon test and adjusted for multiple comparisons by controlling false discovery rate using the Benjamini–Hochberg method. Adjusting results for age, sex, and body mass index of participants increased statistical significance for absolute lymphocyte count (ALC) at admission (*P* = .0337) and ALC nadir (*P* = .0127). See Supplementary Table 2 for details. Abbreviations: ALC, absolute lymphocyte count; ALT, alanine aminotransferase; AST, aspartate aminotransferase; CFR, children fully recovered from coronavirus disease 2019; COVID-19, coronavirus disease 2019; CRP, C-reactive protein; F, female; M, male; MIS-C, multisystem inflammatory syndrome in children; proBNP, prohormone B-type natriuretic peptide; SAC, severe acute coronavirus disease 2019 in children; WBC, white blood cell count.

Between 10 April and 10 July 2020, 19 pediatric MIS-C and 18 SAC patients were recruited at Johns Hopkins Hospital (JHH) and enrolled into the Clinical Characterization Protocol for Severe Infectious Diseases (CCPSEI) cohort as previously described [2] (Figure 1*A*, Table 1). SARS-CoV-2 infection diagnosis was considered confirmed with a positive nucleic acid test (NAT); negative NAT with positive serology; or negative NAT and serology, but exposure to a confirmed COVID-19 contact within 1 month of admission. Seven participants with SAC received oxygen; others were hospitalized with dehydration, tachycardia, and/or severe pain secondary to COVID-19, common COVID-19 symptoms because pulmonary symptoms were less frequent in children in the beginning of the pandemic. Participants were considered to have MIS-C based on the Centers for Disease Control and Prevention case definition at the time [22]: individuals aged <21 years with fever, laboratory evidence of inflammation, severe illness requiring hospitalization, involvement of >2 organ systems, no alternative plausible diagnoses, and positive for current/recent SARS-CoV-2 infection by real-time polymerase chain reaction (PCR), serology, or antigen test; or exposure to a suspected/confirmed COVID-19 case within 4 weeks of symptom onset. Medical records were reviewed by blinded, independent clinicians to classify children. Children fully recovered (CFR) from mild COVID-19 3–9 months postdiagnosis were recruited from the previously described Outpatient SARS-CoV-2 Mild and Asymptomatic Immune Response and Transmission (OutSMART) study [23]. These studies were approved by the Johns Hopkins Medicine Institutional Review Board (IRB00259948 and IRB00245545). Last, we included prepandemic plasma samples from 35 HCs obtained at outpatient visits under a protocol (NCT02179151) and approved by the National Institutes of Health Clinical Center IRB. Parents or guardians of the study subjects provided informed consent. Longitudinal samples were obtained from a subset of recovered hospitalized MIS-C and SAC patients (mean time since hospitalization, 298 ± 59.9 days).

**Table 1.** Study Cohort Demographics, Comorbidities, and Treatments

Characteristic	MIS-C	SAC	CFR
	(n = 19)	(n = 18)	(n = 7)
Demographic characteristics			
Age, y, average (min–max)	9.24 (0.27–17.6)	10.14 (0.03–20.4)	11.0 (5.30–18.0)
Female sex, %	47	55	57
Race, No.			
Asian	1	1	0
Black	5	6	4
White	3	4	2
Native American/Alaska Native	0	0	1
Other	10	7	0
Ethnicity, No.			
Hispanic	11	9	0
Non-Hispanic	8	9	7
Comorbidities			
Average BMI, kg/m^2^	23.65	25.38	18.73
Average BMI percentile	87.02	79.66	60.00
Chronic condition, No. (%)^a^	5 (26)	13 (72)	0 (0)
Immunocompromising condition, No. (%)	0 (0)	3 (17)	0 (0)
ICU characteristics			
ICU hospitalization, No. (%)^b^	15 (79)	7 (39)	NA
Supplemental oxygen, No.	8	7	NA
Pressors, No.	8	0	NA
Treatment characteristics, No.^c^			
IVIG	14	1	NA
Steroids	6	1	NA
Remdesivir	2	1	NA
Convalescent plasma	0	2	NA

Abbreviations: BMI, body mass index; CFR, children fully recovered from coronavirus disease 2019; ICU, intensive care unit; IVIG, intravenous immunoglobulin; MIS-C, multisystem inflammatory syndrome in children; NA, not applicable; SAC, severe acute coronavirus disease 2019.

^a^Chronic conditions include the following: participants with MIS-C: asthma (n = 3), chromosomal/genetic syndromes (n = 2), developmental delays (n = 2), chronic lung disease (n = 1). Participants with SAC: asthma (n = 3), chromosomal/genetic syndromes (n = 2), sickle cell disease (n = 1), chronic pulmonary disease (n = 1), type 2 diabetes mellitus (n = 1), prematurity (n = 1), seizures (n = 1).

^b^Immunocompromised conditions include the following: Participants with SAC: myelodysplastic syndrome (n = 1), hematopoietic stem cell transplantation (n = 1), liver transplant recipient (n = 1).

^c^Patients may have received >1 treatment.

### Specimen Collection

Blood was collected in acid citrate dextrose tubes, and plasma was isolated and stored at −80°C. Peripheral blood mononuclear cells (PBMCs) were isolated within 24 hours of blood collection, as previously described [24], and stored in liquid nitrogen.

### Serum Cytokine and Chemokine Profiling

Serum cytokines and chemokines, including EM-related cytokines (IL-3, IL-27, G-CSF, and TPO), were measured using the Meso Scale Discovery (MSD) V-plex 30 kit following the manufacturer's instructions. Data were acquired on a MESO QuickPlex SQ 120, as previously described [2].

### Immunometabolic Ex Vivo Flow Cytometry Staining

Due to limited blood draw volume in children, we obtained sufficient PBMCs from 12 MIS-C and 11 SAC participants to perform flow cytometry. PBMCs were used for phenotypic and metabolic assessment (Supplementary Table 1, Supplementary Figure S2) as previously described [25] and detailed in the Supplementary Methods.

### Cell-Free DNA Quantification

Plasma sample processing, quantitative PCR, standard curve calculations, and cfDNA concentration calculations were performed and are detailed in the Supplementary Methods. Subsequently, we performed bisulfite conversion, library construction, validation, and sequencing, further described in the Supplementary Methods [26].

### Statistical Analysis

**Figure 2.**
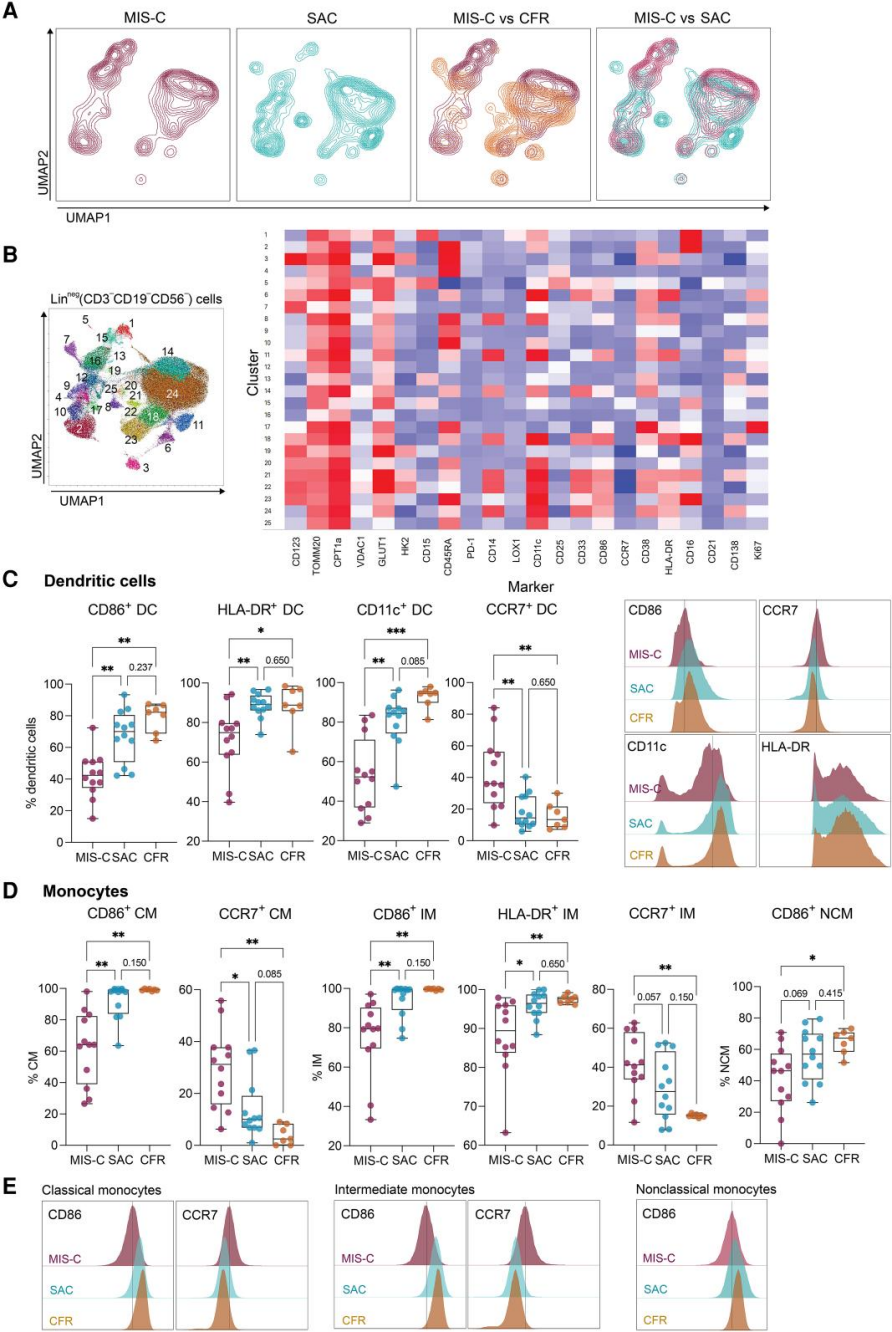
Antigen-presenting cells exhibit an altered phenotype in multisystem inflammatory syndrome in children (MIS-C). *A*, Uniform manifold approximation and projection (UMAP) projection of lineage-negative (CD3^−^CD19^−^CD56^−^) cells in children with MIS-C, severe acute coronavirus disease 2019 (SAC), and children fully recovered from coronavirus disease 2019 (CFR). *B*, XShift algorithm identified 25 myeloid clusters on the UMAP. Heatmap of mean fluorescence intensity (MFI) of markers expressed in each cluster. *C*, Expression of CD86, HLA-DR, CD11c, and CCR7 on dendritic cells (DCs). Wilcoxon test, adjusted for multiple comparisons by controlling false discovery rate (FDR) using Benjamini–Hochberg method. *P < .05, **P < .01, ***P < .001. MFI plots of CD86, CCR7, HLA-DR, and CD11c expression on DCs. *D*, Expression of CD86, HLA-DR, and CCR7 on classical monocytes (CM), intermediate monocytes (IM), and nonclassical monocytes (NCM). Wilcoxon test, adjusted for multiple comparisons by controlling FDR using Benjamini–Hochberg method. *P < .05, **P < .01. *E*, MFI plots of CD86, CCR7, and HLA-DR expression on monocyte subtypes. Adjusting results for age, sex, and body mass index did not alter these results. See Supplementary Table 3 for details.

**Figure 3.**
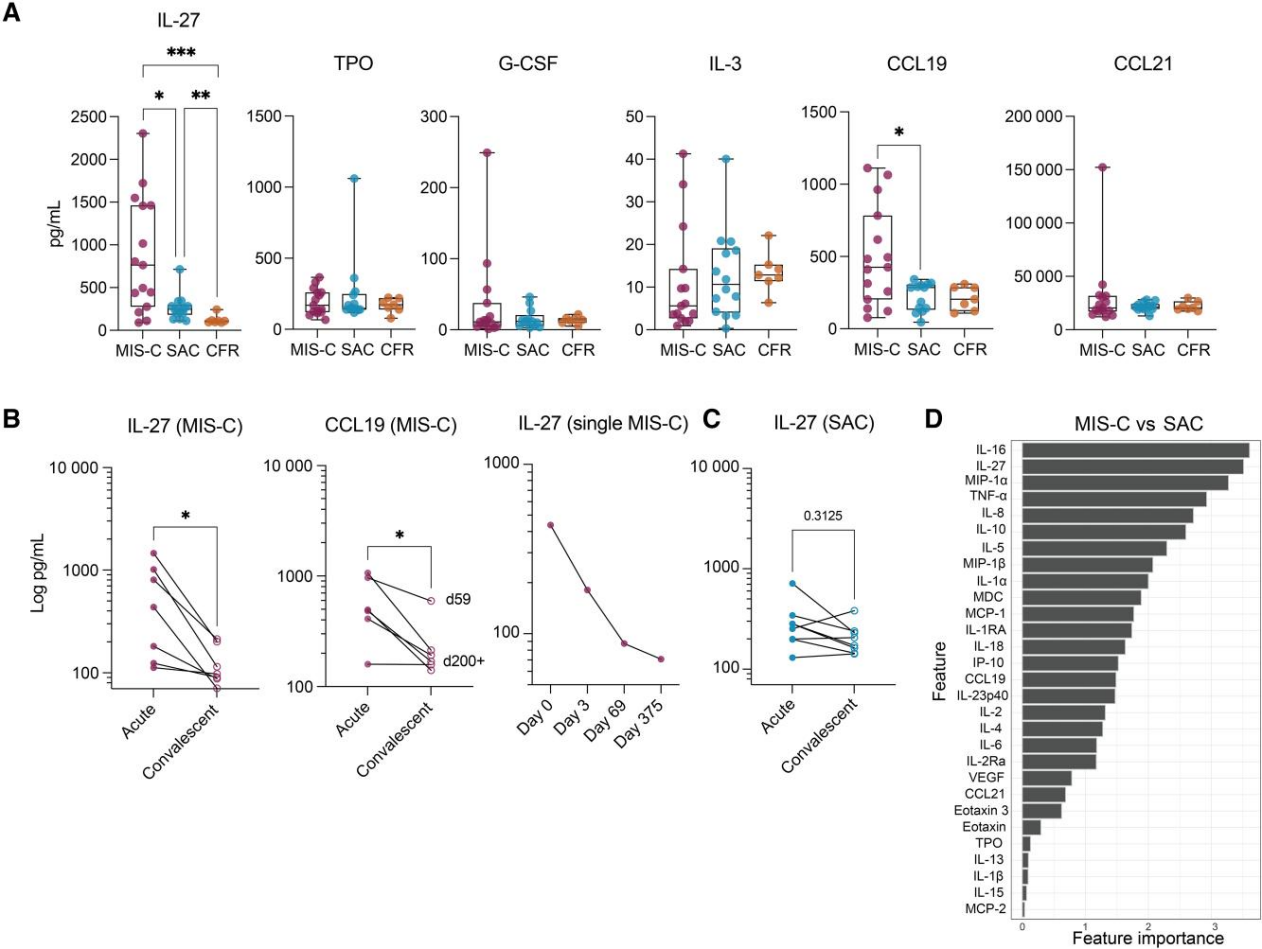
Emergency myelopoiesis signature distinguishes multisystem inflammatory syndrome in children (MIS-C) from acute coronavirus disease 2019 (COVID-19). *A*, Plasma levels of cytokines known to promote hematopoietic stem cell proliferation and differentiation (interleukin [IL] 27, thrombopoietin [TPO], IL-3, and granulocyte colony-stimulating factor [G-CSF]) and the CCR7 receptor ligands CCL19 and CCL21. Only statistically significant differences are shown. Wilcoxon test corrected for multiple comparisons by controlling the false discovery rate using Benjamini–Hochberg method. *P < .05, **P < .01, ***P < .001. Adjusting results for age, sex, and body mass index did not alter the results between MIS-C and severe acute COVID-19 (SAC) groups. See Supplementary Table 4 for details. *B*, Longitudinal comparison of plasma levels of IL-27 in MIS-C and CCL19 in MIS-C (Wilcoxon matched-pairs test, *P < .05); levels of plasma IL-27 in a single individual hospitalized with MIS-C on day 0 (hospitalization), day 3, day 69, and day 375. *C*, Longitudinal plasma IL-27 levels in children hospitalized with acute COVID-19. Wilcoxon matched-pairs test. *D*, IL-27 is the second most important cytokine to distinguish MIS-C and SAC.

**Figure 4.**
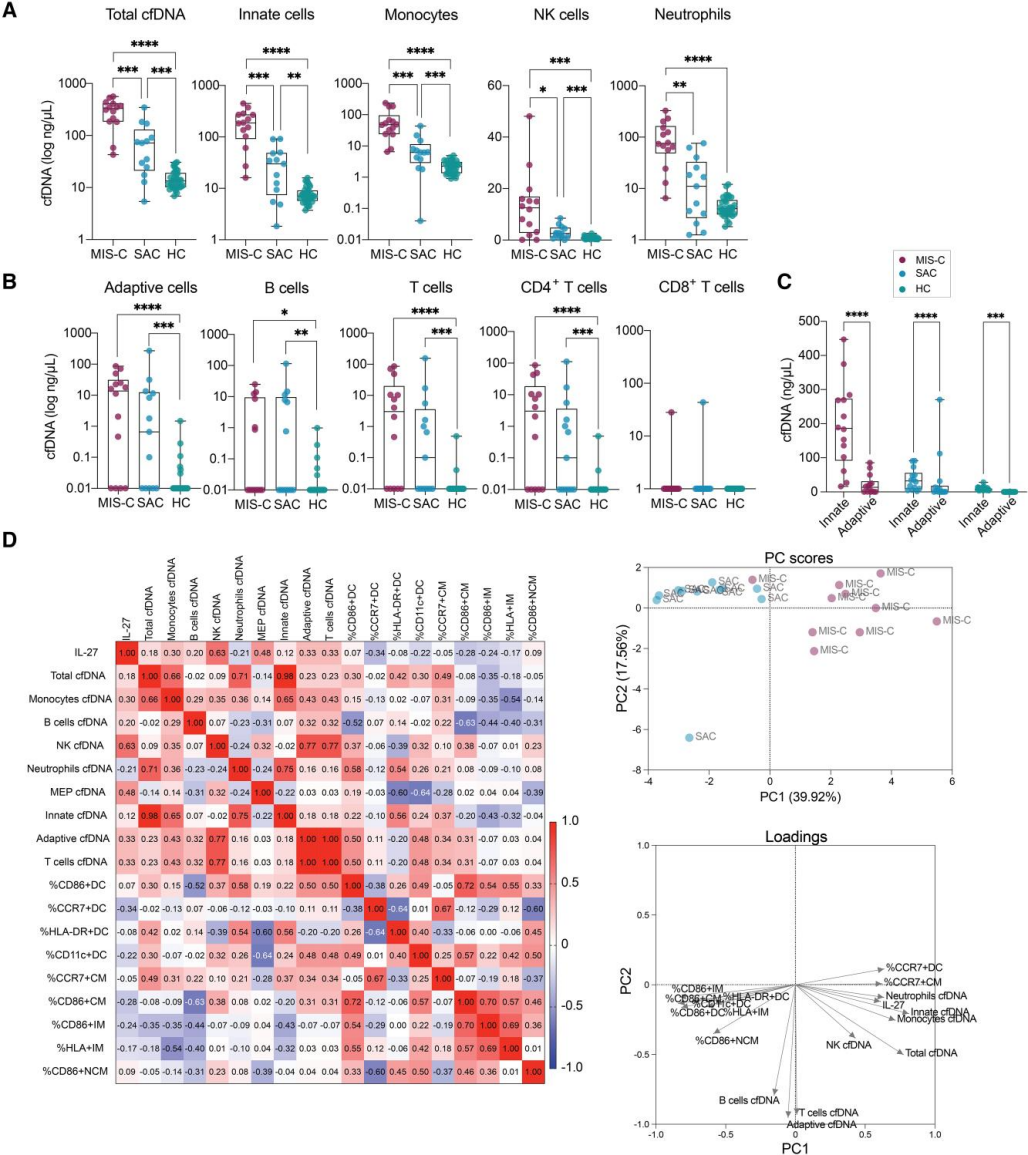
Cell death signatures distinguish multisystem inflammatory syndrome in children (MIS-C) from acute coronavirus disease 2019 (COVID-19) and innate immune cells contribute significantly to elevated cell-free DNA (cfDNA) levels in MIS-C. *A* and *B*, Total cfDNA levels, and levels of cfDNA derived from innate and adaptive immune cells in MIS-C, severe acute COVID-19 (SAC), and 35 pediatric healthy controls (HCs) with pre–COVID-19 pandemic plasma samples. Significance was tested using Wilcoxon test and adjusted for multiple comparisons by controlling the false discovery rate (FDR) using Benjamini–Hochberg method, *P < .05, **P < .01, ***P < .001, ****P < .0001. We redid the analysis without outliers (identified by Grubb test), which yielded identical results. Adjusting results for age, sex, and body mass index did not alter the results (see Supplementary Table 5 for details on *P* values). *C*, All patients exhibited significantly lower levels of cfDNA derived from adaptive immune cells compared to innate immune cells, but this was most significant for MIS-C patients (*P* = 2.628e-13) compared to SAC (*P* = .0001) and HC (*P* = .0075). Significance was tested using Wilcoxon test, adjusted for multiple comparisons by controlling FDR using Benjamini–Hochberg method. ***P < .001, ****P < .0001. *D*, Spearman correlation plot of interleukin 27, emergency myelopoiesis signature on dendritic cells and monocytes, and plasma cfDNA, and principal component (PC) analysis of parameters included in the Spearman correlation.

**Figure 5.**
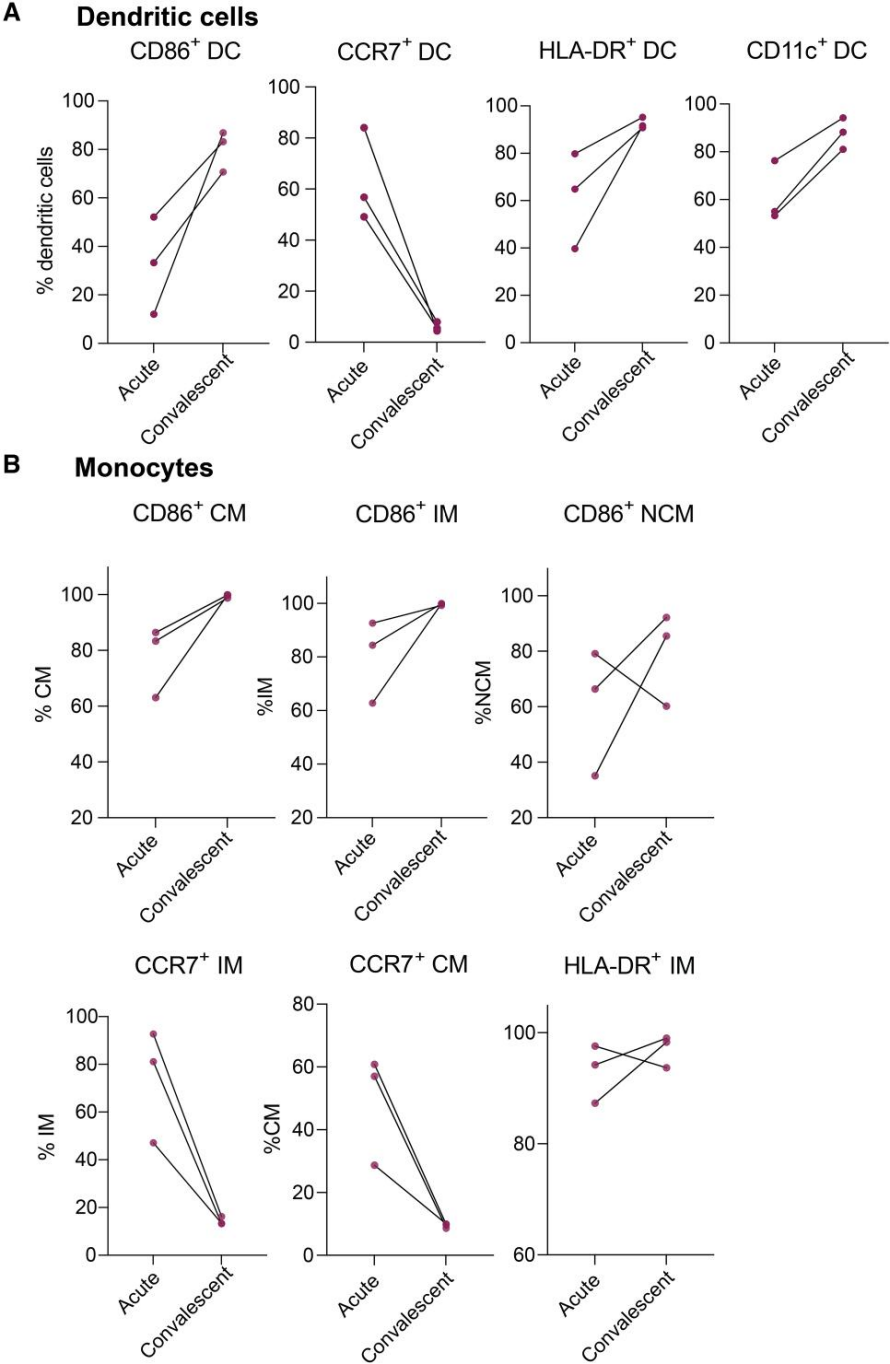
Altered antigen-presenting cell phenotype returns to normal with resolution of multisystem inflammatory syndrome in children. *A* and *B*, CD86, CCR7, HLA-DR, and CD11c expression levels on dendritic cells, and CD86, CCR7, and HLA-DR expression levels on monocytes return to normal levels upon recovery. Significance was tested using Wilcoxon test, adjusted for multiple comparisons by controlling false discovery rate using Benjamini–Hochberg method. All relationships were nonsignificant, presumably due to small sample sizes. Abbreviations: DC, dendritic cell; IM, intermediate monocyte; NCM, nonclassical monocyte.

**Figure 6.**
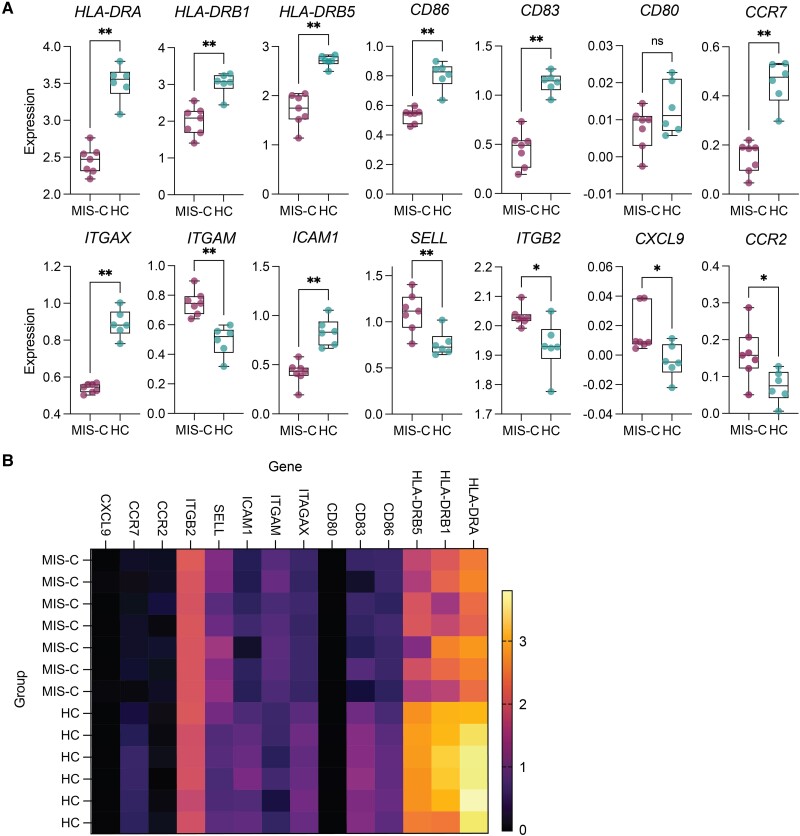
Single-cell RNA sequencing analysis of publicly available dataset (GSE166489). *A*, Expression values of genes of interest comparing multisystem inflammatory syndrome in children (MIS-C) and healthy control (HC) groups. Significance tested using Wilcoxon test with Benjamini–Hochberg method, *P < .05, *P < .01, ns = not significant. *B*, Heatmap of genes plotted in *A*.

Statistical calculations were performed in GraphPad Prism 9 and RStudio version 4.2.2 software. Data are shown as mean ± standard error of the mean unless otherwise indicated in figure legends. Differences were considered statistically significant at a *P* value < .05. We used the Wilcoxon test for *P* value calculations and the Benjamini–Hochberg method for *P* value adjustment in Figures 1–6. We utilized a random forest machine learning model with R packages caret [18] and randomForest [27] to identify the most predictive features for MIS-C. For the single-cell RNA sequencing data analysis, we used data and annotations provided in [12] to obtain the gene expression of myeloid cells. Gene counts were aggregated within each cell type, forming pseudobulk samples per cell type per patient. Pseudobulk counts were normalized using the NormalizeData function in the Seurat R package [20]. Expression of EM genes was obtained, and a 2-sided Mann–Whitney *U* test compared gene expression between MIS-C patients and HCs in myeloid cells. *P* values underwent false discovery rate transformation for multiple testing adjustment via the Benjamini–Hochberg procedure [22]. Additional details are described in the Supplementary Methods [12, 27–31]. To mitigate age, sex, and body mass index (BMI) effects, we employed linear regression with these variables as covariates. The *P* value of the variable of interest's coefficient was determined, adjusted for age, sex, and BMI. Subsequently, we applied the Benjamini–Hochberg method for *P* value correction (Supplementary Tables 2–5).

## RESULTS

### Clinical Parameters in MIS-C and Patients With Severe Acute COVID-19

While the mechanisms underlying MIS-C are unclear, several clinical parameters have been reported to distinguish MIS-C from SAC [1, 7, 8]. In our study, no clinical parameters were significantly different between SAC and MIS-C when adjusted for multiple comparisons. However, MIS-C patients tended to exhibit decreased lymphocyte and platelet counts at nadir, along with higher white blood cell counts at peak (Figure 1*B*). Inflammation biomarkers, including C-reactive protein, D-dimer, and prohormone B-type natriuretic peptide were elevated in MIS-C patients compared to SAC, although not reaching statistical significance (Figure 1*C*).

To further compare proinflammatory profiles, we measured 36 cytokines and chemokines using the MSD platform. The previously published results [2] showed elevated levels of several analytes in MIS-C compared to individuals with SAC. Considering these laboratory abnormalities and the absence of clear distinguishing patterns between MIS-C and SAC, we aimed to explore the underlying mechanisms.

### MIS-C Is Associated With Alterations in the Myeloid Cell Compartment

We first examined immune aberrations of PBMCs collected from MIS-C, SAC, and CFR participants using a recently published 32-color flow cytometry panel with modifications (Supplementary Table 1) [25]. This panel includes 24 surface and 7 intracellular molecules and can distinguish and characterize over 20 different cellular populations.

Given the general lymphopenia and previous reports identifying T-cell activation in MIS-C [32, 33], we performed in-depth analysis of T-cell immunometabolic profiles. T cells in MIS-C patients were decreased in frequency compared to CFR, but not SAC (Supplementary Figure 1*A*). Consistent with other studies, MIS-C T cells exhibited activation and proliferation, indicated by the frequency of programmed death 1 (PD-1)^+^, HLA-DR^+^ and CD38^+^ coexpression, and Ki67^+^ nonnaive T cells (Supplementary Figure 1*A*) [32, 33]. This was especially apparent in CD8^+^ T cells in MIS-C but did not distinguish MIS-C and SAC patients (Supplementary Figure 1*A* and 1*B*). Both MIS-C and SAC T cells expressed increased levels of CCR7 compared to CFR, suggesting heightened migration out of peripheral blood (Supplementary Figure 1*C*). Naive CD8^+^ T cells expressed comparable levels of CCR7 in MIS-C and SAC individuals, but nonnaive CD8^+^ and CD4^+^ T cells expressed more CCR7 in MIS-C (Supplementary Figure 1*C*). Additionally, we report increased expression of carnitine palmitotransferase 1a, the rate-limiting enzyme of fatty acid oxidation (FAO), particularly in PD-1^+^CD8^+^ T cells of MIS-C patients (Supplementary Figure 1*D*). PD-1 expression on activated T cells has been shown to prevent glycolysis and enhance FAO, supporting the activated T-cell phenotype [34]. Thus, despite lymphopenia, T cells in MIS-C and SAC appear activated, proliferating, and actively migrating from the periphery.

We then scrutinized the myeloid cell compartment due to significant clustering differences between MIS-C, SAC, and CFR observed on the uniform manifold approximation and projection (Figure 2*A*). XShift, an unsupervised clustering algorithm that uses k-nearest neighbors density estimation, was then applied to myeloid cells, and identified 25 distinct clusters (Figure 2*B*) [35]. Clusters 1 (polymorphonuclear myeloid-derived suppressor cells [MDSC]), 14 (monocytic MDSC), and 15 (neutrophils) did not appear in SAC and were specific to MIS-C, while clusters 8 (activated classical monocytes [CMs]), 10 (mature dendritic cells [DCs]), and 11 (CMs) were enriched in patients with SAC (Figure 2*A* and 2*B*). This unbiased approach confirmed robust differences between MIS-C and SAC within the myeloid compartment, characterized by increased frequencies of MDSCs and granulocytes in MIS-C and by mature monocytes and mature DCs in SAC.

Manual gating was then applied to evaluate surface markers traditionally expressed on myeloid cells. Professional APCs, including monocytic and plasmacytoid DCs, expressed significantly less CD86, HLA-DR, and CD11c but more CCR7 in MIS-C compared to SAC and CFRs, both in frequency and mean fluorescence intensity (Figure 2*C*). Similarly, CMs and intermediate monocytes (IMs) expressed less CD86 in MIS-C (Figure 2*D*). Additionally, CMs and IMs in MIS-C expressed a trend toward increased CCR7 expression, and IMs in MIS-C expressed significantly less HLA-DR compared to the other groups (Figure 2*D*). In summary, we report low expression of costimulatory molecules on APCs and increased surface expression of G-protein coupled receptor CCR7, which classically promotes migration out of the periphery to lymphoid tissues and is a typical marker of naive and central memory T cells and of myeloid cells during infection and inflammation. Combined, expression levels of these costimulatory and trafficking molecules on APCs distinguished MIS-C from SAC.

### MIS-C Patients Exhibit an Emergency Myelopoiesis Signature

Reduced expression of costimulatory molecules on APCs in MIS-C has been associated with the phenomenon of EM, previously reported in the acute phase of severe COVID-19 in hospitalized adults [13–15]. Recent studies identified the proinflammatory cytokine IL-27 as one of the strongest inducers of continuous HSC expansion [17–21]. Therefore, we measured cytokines that may have induced EM in our cohort. IL-27 was significantly elevated in MIS-C, distinguishing it from both SAC and CFR (Figure 3*A*). Because we observed elevated levels of CCR7 receptor levels on T cells and APCs in MIS-C, we simultaneously measured plasma levels of the 2 chemokine ligands of CCR7, CCL19, and CCL21. Plasma levels of CCL19, but not CCL21, were significantly elevated in MIS-C compared to SAC (Figure 3*A*).

Longitudinal analysis of IL-27 and CCL19 plasma levels in 7 MIS-C individuals revealed significant decreases over time regardless of time since hospitalization (range, 59–403 days) (Figure 3*B*), suggesting that EM is an acute phenomenon rather than an indicator of increased MIS-C risk. In a single patient with MIS-C treated with intravenous immune globulin, methylprednisolone, anakinra, and aspirin, we detected a significant drop in IL-27 levels after treatment initiation through recovery (Figure 3*B*). Conversely, there was no significant decrease between acute and convalescent plasma IL-27 levels in SAC patients (Figure 3*C*).

Plasma levels of 42 cytokines and chemokines were included in a predictive analysis to identify parameters that distinguish MIS-C and SAC. After IL-16, IL-27 was the second most important feature differentiating the 2 groups (Figure 3*D*). While the role of IL-16 in MIS-C is not understood, it has been shown to induce migration of T cells to sites of inflammation and tissue damage [36]. Therefore, IL-16 could contribute to lymphocyte egress out of peripheral blood observed in MIS-C. However, more evidence is needed to support this hypothesis.

### Myeloid Cells in MIS-C Patients Exhibit Increased Cell Death That Correlates With the EM Phenotype

MIS-C patients exhibit lymphopenia, but it remains unknown whether due to cell death or migration to inflamed sites. Moreover, although myeloid cells exhibited heightened expression of surface markers associated with immature states, it is uncertain whether this is linked to increased innate cell death. Thus, we measured plasma cfDNA levels, an organ-specific marker of tissue injury and cell death that can serve as a dynamic biomarker of inflammation, because healthy individuals have very low circulating cfDNA levels [37–40]. Additionally, cfDNA retains its tissue-specific DNA methylation signature, providing insights into biological pathways related to disease pathology [26, 41–43].

We utilized quantitative PCR to measure cfDNA levels in MIS-C, SAC, and a group of 35 HCs with pre–COVID-19 pandemic plasma samples. Total cfDNA levels were significantly elevated in MIS-C than in SAC and HCs, indicating greater tissue injury in MIS-C (Figure 4*A*). We then performed whole-genome bisulfite sequencing and utilized a library of tissue-specific DNA methylation signatures to assign the cfDNA sources [44]. Interestingly, the difference in total cfDNA levels was mainly attributed to cfDNA derived from innate immune cells (Figure 4*A*). Compared to SAC, MIS-C had higher cfDNA levels from monocytes, neutrophils, and natural killer cells (Figure 4*A*). Conversely, cfDNA derived from adaptive immune cells did not differentiate MIS-C from SAC, with only a few individuals in each group showing increased levels (Figure 4*B*). Innate cell-derived cfDNA levels were >8-fold higher than adaptive cell-derived cfDNA in MIS-C individuals (Figure 4*C*). While adaptive cells also played a role in the increase of cfDNA in MIS-C and SAC, a significant portion, especially in MIS-C, originated from the innate immune cell compartment.

A positive correlation trend was observed between plasma levels of IL-27 and cfDNA levels in MIS-C patients, indicating that the increased EM signature is associated with cell death and reduced frequency of mature myeloid cells in peripheral blood (Figure 4*D*). Conversely, the EM signature, characterized by low expression of CD86 and HLA-DR on APCs tended to be inversely correlated with IL-27 levels, suggesting decreased counts of mature APCs in presence of increased IL-27 plasma levels. Principal component analysis, including IL-27, EM signature, and cfDNA levels, clearly differentiated the MIS-C and SAC groups (Figure 4*D*).

### Altered APC Phenotype Returns to Normal With MIS-C Symptom Resolution

Longitudinal PBMC samples from 3 hospitalized MIS-C patients were assessed to determine if the EM signature was present only with severe symptoms. Despite limited sample size, flow cytometry analysis revealed that the expression of CD86, HLA-DR, CCR7, and CD11c on DCs returned to normal levels upon recovery for each participant (Figure 5*A*). Similar trends were observed for monocytes (Figure 5*B*), indicating that following MIS-C resolution, the blood cell reservoir is replenished with mature populations of APCs and other myeloid cells.

### Single-Cell RNA Sequencing Analysis Supports EM

Last, we assessed expression of genes associated with EM in children with MIS-C and HCs using publicly available scRNAseq dataset (GSE166489) [12]. Consistent with our flow cytometry findings, myeloid cells in MIS-C showed significant downregulation of transcripts involved in antigen presentation, including *HLA-DRA*, *HLA-DRB1*, *HLA-DRB5*, *CD83*, *CD86*, and *ICAM1*, and the DC marker *ITAGAX* (CD11c) (Figure 6*A* and 6*B*). We also surveyed gene transcripts involved in cell adhesion and migration, finding that *CCR7* gene was downregulated in MIS-C despite our flow results showing CCR7 upregulation on APCs and increased plasma levels of CCL19 (Figures 2*C*, 2*D*, and 3*A*). This highlights the need to use both flow cytometry and scRNAseq data to define cell phenotypes. Conversely, cell adhesion molecules *SELL* (CD62L), *ITGAM* (CD11b), and *ITGB2* (CD18), and cell migration molecules *CCR2* and *CXCL9* were upregulated in MIS-C (Figure 6*A* and 6*B*), indicating myeloid cell recruitment from blood to tissues during inflammation [45]. The scRNAseq findings were consistent with our flow cytometry results and validate the transcriptional profiles associated with alterations in the myeloid compartment observed in MIS-C.

## DISCUSSION

This study identified innate immune alterations in MIS-C, offering insights into the dysregulated immunity that distinguishes the syndrome from SAC. High-dimensional flow cytometry, cfDNA quantification, and plasma cytokine/chemokine measurements revealed an EM immune signature and significant innate immune cell death unique to MIS-C.

Clinical parameters alone provide limited mechanistic insight into MIS-C pathogenesis. Therefore, we employed flow cytometry to identify potential key cell types and pathways critical in driving MIS-C. Decreased expression of antigen presentation markers in MIS-C compared to SAC and CFR represented the most significant difference observed in our flow cytometry analysis. Following antigen exposure, professional APCs undergo a maturation process to more efficiently present antigen. However, the profound decreases in HLA-DR and CD86 expression on APCs suggest low antigen-presenting capacity and recent emigration from the bone marrow. This was supported by scRNAseq data (GSE166489) showing downregulation of transcripts involved in antigen presentation in MIS-C compared to HCs. Upon recovery from MIS-C, DCs and monocytes regained normal surface expression levels of HLA-DR, CD86, CCR7, and CD11c, suggesting replenishment of mature myeloid cells after MIS-C resolution.

The robust immature myeloid cell signature in MIS-C patients prompted us to investigate the phenomenon of EM. EM restores blood cell homeostasis and promotes tissue repair in response to low myeloid cell counts during infection and inflammation [17–21]. In contrast to significant lymphopenia, MIS-C patients did not exhibit reduced innate cell counts, strengthening our hypothesis that EM replenishes the myeloid cell reservoir, negating significant decreases in innate immune cell populations. MIS-C patients in our study exhibited significantly elevated plasma levels of IL-27, the strongest inducer of continuous HSC expansion [17, 19, 46]. IL-27 levels decreased upon MIS-C resolution, supporting the role of EM in acute MIS-C. Similarly, IL-27 levels from a single individual treated in the intensive care unit significantly decreased after initiation of effective therapy. Consistent with our results, upregulation of the *IL27* gene differentiated MIS-C from patients hospitalized with febrile illness in South Africa [47]. Understanding the role of EM in MIS-C will require longitudinal research to determine if it predicts critical illness [15].

The causes of innate immune cell death in MIS-C are unknown. Given the remarkably elevated cfDNA levels in MIS-C, cfDNA fragments potentially trigger cytokine production by myeloid cells, including IL-27. Additionally, quiescent HSCs can be activated to proliferate into myeloid cell precursors by DAMPs such as cfDNA [18]. Whether cfDNA fragments activate HSCs to undergo EM remains to be investigated. However, the correlation between EM signatures, IL-27, and cfDNA levels in MIS-C patients potentially supports this notion.

MIS-C patients experience lymphopenia [4, 33], but is has been unclear whether this is due to cell death or migration to sites of inflammation. Our findings suggest that lymphoid cell death is not a significant contributor to lymphopenia in MIS-C. Instead, we observed increased peripheral T-cell proliferation, activation, and evidence of migration out of peripheral blood (CCR7 upregulation), possibly to lymph nodes and affected organs. This provides novel evidence that T cells in MIS-C undergo efflux from peripheral blood that is not driven by T-cell death.

Study limitations include a small dataset due to limited MIS-C blood donors and the absence of prepandemic PBMCs from HCs. Enrollment of MIS-C patients at JHH ceased in late 2020; hence, our findings reflect responses to prevaccination and pre-Omicron strains of SARS-CoV-2. The use of a scRNAseq dataset (GSE166489) restricted comparisons to MIS-C and HCs, as no MIS-C versus SAC datasets were available, potentially obscuring important distinctions between MIS-C and SAC.

In conclusion, we observed a profoundly immature phenotype of peripheral APCs in MIS-C, significant innate immune cell death, and increased migration to lymphoid tissues. This, coupled with unchanged myeloid cell frequencies in the periphery of MIS-C patients, supports continuous replenishment of myeloid cells by proliferating HSCs via EM. We detected significantly elevated levels of IL-27 in MIS-C, the cytokine previously shown to be a major driver of EM. Lymphopenia in MIS-C was not attributed to T-cell death, and our data indicate T-cell egress from the peripheral blood, presumably to tissues. Our results highlight that dysregulation of innate immune cells is a primary driver of MIS-C pathogenesis, and that EM and innate immune cell death distinguish MIS-C from SAC. Further studies on these unique signatures could improve MIS-C identification and early treatment.

## Supplementary Data

Supplementary materials are available at *The Journal of Infectious Diseases* online (http://jid.oxfordjournals.org/). Supplementary materials consist of data provided by the author that are published to benefit the reader. The posted materials are not copyedited. The contents of all supplementary data are the sole responsibility of the authors. Questions or messages regarding errors should be addressed to the author.

## Supplementary Material

jiae032_Supplementary_Data

## Notes

***Acknowledgments.*** The MIS-C and SAC specimens from COVID-19 patients used for this study were part of the JHH Biospecimen Repository, which is based on the contribution of patients, their families, and research teams. We acknowledge the technicians who processed blood at the Viral Hepatitis Center.

***Author contributions.*** K. R., E. A. T., A. H. K., and A. L. C. conceptualized the study and designed methodology. K. R., T. E. A., and T. S. J. performed experimental assays and acquired data. H. J., S. A.-E., and A. L. C. acquired funding. O. G., N. P. A., A. A. R. A., Y. C. M., and D. P. recruited participants and provided samples and clinical data. K. R., T. E. A., A. H. K., Y. W., W. Z., and H. J. performed statistical analyses. K. R. and A. L. C. wrote the original manuscript. All authors read and provided edits to the manuscript and approved the final submission.

***Data availability.*** Data are available in the Supplementary Materials or upon reasonable request.

***Financial support*.** This work was supported by the National Institutes of Health (grant number U54CA260492 to A. L. C.; award number K08AI156021 to A. H. K.; award number T32AI052071 to N. P. A.; and grant number R01HG009518 to H. J.); by intramural research funds of the National Heart, Lung, and Blood Institute (grant number ZIAHL006268) and the Lasker Clinical Research Fellowship Program (to S. A.-E.; 1-Si2-HL147625); the Bauernschmidt Committee of the Eudowood Board at Johns Hopkins School of Medicine, and Johns Hopkins University Provost Research Grant (to N. P. A.).
