# Supplementary material for: Emergency Myelopoiesis Distinguishes Multisystem Inflammatory Syndrome in Children From Pediatric Severe Coronavirus Disease 2019
Source: J Infect Dis. 2024 Jan 31;230(2):e305–17. doi: 10.1093/infdis/jiae032 (PMC11326850; doi:10.1093/infdis/jiae032)
Supplement: jiae032_Supplementary_Data [file jiae032_supplementary_data.docx]

**Emergency myelopoiesis distinguishes multisystem inflammatory syndrome in children from pediatric severe COVID-19**

Katerina Roznik, Temesgen E. Andargie, T. Scott Johnston, Oren Gordon, Yi Wang, Nadine Peart Akindele, Deborah Persaud, Annukka A. R. Antar, Yukari C. Manabe, Weiqiang Zhou, Hongkai Ji, Sean Agbor-Enoh, Andrew H. Karaba, Elizabeth A. Thompson, and Andrea L. Cox

**SUPPLEMENTARY MATERIALS**

**SUPPLEMENTARY METHODS**

**Immuno-metabolic *ex vivo* flow cytometry staining**

PBMCs were used for phenotypic and metabolic assessment. Surface and intracellular antibodies used are outlined in Table S1. Thawed PBMCs were washed in phosphate-buffered saline (PBS) and stained for viability with Biolegend Live/Dead Zombie NIR Fixable Viability Dye and BD Biosciences Fc Block^TM^ for 15 min at room temperature (RT). Cell surface staining was performed in 100 μL of 20% BD Horizon^TM^ Brilliant Stain Buffer and PBS with surface stain antibodies for 20 min at RT. Subsequently, cells were fixed and permeabilized with eBioscience^TM^ FOXP3/Transcription Factor Staining kit 1x Fixation/Permeabilization reagent for 20 min at RT and washed. Intracellular staining (ICS) was performed in 100 μL 1x Permeabilization/Wash buffer with ICS antibody cocktail for 30 min at RT. Cells were washed once with Permeabilization/Wash buffer, resuspended in 1% PFA and run on a 4 laser (16UV-16V-15B-8R) Cytek Aurora spectral flow cytometer.

**Flow cytometry analysis**

FCS files generated by the flow cytometer were analyzed using FlowJo v10 (10.8.1) software. Representative manual gating strategy is detailed in Figure S2. After pre-processing using biexponential transformation, T cells and lineage negative cells were exported separately from FlowJo for further analysis in FlowJo. Data were then analyzed using the XShift algorithm, AutoGateCategorical and ClusterExplorer plugins for unsupervised clustering and visualized with Uniform Manifold Approximation and Projection (UMAP). Finally, data were then reorganized and exported for further statistical analyses using R and Prism GraphPad.

**Cell-free DNA quantification**

To measure circulating cfDNA level, plasma samples were thawed and further centrifuged at 16,000× g for 5 min at 4 °C to remove residual debris. cfDNA extraction was performed from lambda DNA spiked-in 1 mL plasma using Biamp Circulating Nucleic Acid Kit (QIAGEN, Germany) and the quality checked using Cell-free DNA ScreenTape assay (Agilent Technologies). Quantitative PCR (qPCR) were performed to measure the concentration of cfDNA and extraction efficiency using short Alu115 fragments and lambda DNA primers (Promega), respectively. Briefly, 10 μL qPCR reactions containing 5 μL SYBR Green Supermix (Bio-Rad), 2 μL nuclease-free water, 1 μL primer pair and 2 μL cfDNA template (1:10 diluted) were prepared in triplicate and run on QuantStudio 3 qPCR cycler (Applied Biosystems). The thermal cycling conditions used were as follows: initial denaturation at 95°C for 5 minutes, 35 cycles of 95°C for 15 seconds and 60°C for 1 min. A standard curve with 10-fold serial dilutions (1.1 ng to 1.1 × 10^–5^ ng) were used to calculate the concentrations. The amount of quantified lambda DNA was divided by the spiked-in lambda DNA to measure extraction efficiency. The total concentration of cfDNA per milliliter of plasma (ng/mL) were obtained as follow: mean values of Alu115 triplicates data (ng/µL) × extraction elution volume (µL) x dilution factor ÷ plasma volume (µL) ÷ extraction efficiency.

Bisulfite conversion was carried out on 5 to 50 ng of extracted cfDNA using EZ DNA methylation-Gold kit (Zymo Research) followed by dual-indexed sequencing library construction using the Accel-NGS Methyl-Seq DNA Library Kit (Swift Biosciences) for whole-genome bisulfite sequencing. The quality and concentration of the library was determined using high-sensitivity D1000 ScreenTape (Agilent Technologies) and Quant-iT PicoGreen dsDNA assay kit (Life Technologies), respectively. Individual libraries were pooled in equimolar amounts and paired end sequenced (2x100 bp) on Illumina NovaSeq 6000. The quality of sequence reads was checked by FastQC, followed by adapter removal with TrimGalore and aligned to the hg19 human reference genome with Bismark. After alignment, Bismark was used to trim 10 bp from both ends of reads, remove PCR duplicates, post-alignment quality control, and extract cytosine methylation states and calculate all CpGs in individual samples. The relative proportion of cell-specific cfDNA was deconvoluted using the meth_atlas algorithm [17]. To calculate the absolute amount of cell-specific cfDNA per mL of plasma, the estimated cell-type proportions of cfDNA are multiplied by the total cfDNA (ng/mL) concentration.

**Statistical Analyses**

To identify the features that are most predictive of MIS-C, we applied a multivariate analysis based on a machine learning model, random forest, using R packages caret [18] and randomForest [19]. We first trained a random forest model using the 42 cytokines and chemokines as features based on leave-one-sample-out cross-validation for predicting MIS-C vs. AC patients. We then calculated the feature importance based on the mean decrease of accuracy to identify features that are most predictive of MIS-C. For the single-cell RNA sequencing data analysis, scRNA-seq data from PBMC of MIS-C patients and healthy pediatric controls were downloaded from GEO (GSE166489). We used the processed data and the annotation provided in [12] to obtain the gene expression of myeloid cells. The counts for each gene were aggregated within each cell type across the cells to form a pseudobulk sample for each cell type in each patient. Then, pseudobulk counts were normalized using the NormalizeData function in the Seurat R package [20]. To remove the batch effect, the sva function in the sva R package [21] was used to estimate and remove the effect of surrogate variables from the pseudobulk samples. Gene expression of the EM genes was obtained from the pseudobulk sample of each cell type in each patient. To compare the gene expression between MIS-C patients and healthy controls, a two-sided Mann–Whitney U test was applied to each EM gene in the myeloid cells. P-values were transformed to false discovery rate (FDR) to adjust for multiple testing using the Benjamini-Hochberg procedure [22].

**SUPPLEMENTARY TABLES**

**Table S1. Flow cytometry panel for assessing the phenotype of participant PBMCs.**

**Surface**

| **Fluorophore** | **Marker** | **Clone** | **Catalog** | **Vendor** |
| --- | --- | --- | --- | --- |
| Zombie NIR | Viability | n/a | 423106 | BioLegend |
| BV570 | CD33 | WM53 | 303417 | BioLegend |
| BV480 | CD11c | B-ly6 | 566135 | BD Biosciences |
| APC | CD123 | 6H6 | 306012 | BioLegend |
| BV510 | CD25 | M-A251 | 563352 | BD Biosciences |
| BV650 | CCR7 | G043H7 | 353234 | BioLegend |
| BV421 | LOX1 | 15C4 | 358610 | BioLegend |
| BV711 | CD38 | HIT2 | 563965 | BD Biosciences |
| APC-Cy7 | CD19 | SJ25C1 | 363010 | BioLegend |
| APC-Cy7 | CD56 | 5.1H11 | 362512 | BioLegend |
| BV750 | HLA-DR | L243 | 307672 | BioLegend |
| BUV661 | CD3 | UCHT1 | 612964 | BD Biosciences |
| BUV496 | CD4 | SK3 | 612936 | BD Biosciences |
| BUV737 | CD8 | SK1 | 612754 | BD Biosciences |
| BV785 | CD16 | 3G8 | 302046 | BioLegend |
| BUV805 | CD14 | M5E2 | 612902 | BD Biosciences |
| BV605 | CD86 | BU63 | 374214 | BioLegend |
| BUV563 | CD45RA | HI100 | 612926 | BD Biosciences |
| BUV395 | CD15 | H198 | 563872 | BD Biosciences |
| Spark NIR | CD27 | O232 | 302856 | BioLegend |
| PE-Cy5 | CD21 | B-ly4 | 551064 | BD Biosciences |
| PE-Cy5.5* | CD138 | MI15 | 356502 | BioLegend |
| PE-CF594 | KLRG1 | 2F1 | 565393 | BD Biosciences |
| BB790 | IgD | IA6-2 | custom | BD Biosciences |
| BUV615 | PD-1 | EH12.1 | 612991 | BD Biosciences |

**Intracellular**

| **Fluorophore** | **Marker** | **Clone** | **Catalog** | **Vendor** |
| --- | --- | --- | --- | --- |
| Pacific Blue | FOXP3 | 206D | 320116 | BioLegend |
| PE-Cy7 | Ki-67 | Ki-67 | 350526 | BioLegend |
| Alexa Fluor 488 | CPT1a | 8F6AE9 | ab171449 | Abcam |
| Alexa Fluor 680** | HK2 | EPR20839 | ab228819 | Abcam |
| Alexa Fluor 532*** | VDAC1 | 20B12AF2 | ab14734 | Abcam |
| Alexa Fluor 405 | Tomm20 | EPR15581-54 | ab210047 | Abcam |
| Alexa Fluor 647 | GLUT1 | EPR3915 | ab195020 | Abcam |

Notes: * conjugated using PE/Cy5.5 Conjugation Kit - Lightning-Link (ab102899)

** conjugated using DyLight 680 Conjugation Kit (Fast) - Lightning-Link (ab201804)

*** conjugated using Alexa Fluor 532 Antibody Labeling Kit (Thermo Fisher, A20182)

**Table S2. P-values for Figure 1 after covariate analysis adjusting for age, sex, and body mass index of study participants and adjusting for multiple comparisons using Benjamini-Hochberg procedure.**

| **Parameter** | **P-value** |
| --- | --- |
| WBC admission | 0.4776 |
| WBC peak | 0.3472 |
| ALC admission | 0.0337 |
| ALC nadir | 0.0127 |
| Platelets admission | 0.1912 |
| Platelets nadir | 0.0755 |
| CRP admission | 0.4776 |
| CRP peak | 0.2728 |
| D-dimer admission | 0.4537 |
| D-dimer peak | 0.3047 |
| Troponin admission | 0.5498 |
| Troponin peak | 0.4113 |
| ProBNP admission | 0.4776 |
| ProBNP peak | 0.4113 |
| AST | 0.3462 |
| ALT | 0.3880 |
| Creatinine | 0.4113 |
| Ferritin | 0.4113 |
| Neutrophil count (%) | 0.4607 |

**Table S3. P-values for Figure 2 after covariate analysis adjusting for age, sex, and body mass index of study participants and adjusting for multiple comparisons using Benjamini-Hochberg procedure.**

**MIS-C vs. CFR**

| **Parameter** | **P-value** |
| --- | --- |
| CD86 DC | 0.0023 |
| CCR7 DC | 0.0180 |
| HLA-DR DC | 0.1466 |
| CD11c DC | 0.0179 |
| CD86 CM | 0.0056 |
| CCR7 CM | 0.0033 |
| CD86 IM | 0.0023 |
| HLA-DR IM | 0.1069 |
| CCR7 IM | 0.0180 |
| CD86 NCM | 0.0314 |

**MIS-C vs. SAC**

| **Parameter** | **P-value** |
| --- | --- |
| CD86 DC | 0.0172 |
| CCR7 DC | 0.0172 |
| HLA-DR DC | 0.0203 |
| CD11c DC | 0.0303 |
| CD86 CM | 0.0169 |
| CCR7 CM | 0.0196 |
| CD86 IM | 0.0169 |
| HLA-DR IM | 0.0372 |
| CCR7 IM | 0.2439 |
| CD86 NCM | 0.1234 |

**SAC vs. CFR**

| **Parameter** | **P-value** |
| --- | --- |
| CD86 DC | 0.0818 |
| CCR7 DC | 0.4709 |
| HLA-DR DC | 0.9279 |
| CD11c DC | 0.0519 |
| CD86 CM | 0.1170 |
| CCR7 CM | 0.0818 |
| CD86 IM | 0.3366 |
| HLA-DR IM | 0.9279 |
| CCR7 IM | 0.1169 |
| CD86 NCM | 0.0818 |

**Table S4. P-values for Figure 3A after covariate analysis adjusting for age, sex, and body mass index of study participants and adjusting for multiple comparisons using Benjamini-Hochberg procedure.**

**MIS-C vs. SAC**

| **Parameter** | **P-value** |
| --- | --- |
| IL-27 | 0.0463 |
| TPO | 0.4234 |
| G-CSF | 0.3393 |
| IL-3 | 0.3883 |
| CCL21 | 0.3393 |
| CCL19 | 0.0463 |

**Table S5. P-values for Figure 4 after covariate analysis adjusting for age, sex, and body mass index of study participants and adjusting for multiple comparisons using Benjamini-Hochberg procedure.**

**MIS-C vs. HC**

| **Parameter** | **P-value** |
| --- | --- |
| Total cfDNA | 1.747e-13 |
| Innate cells | 9.930e-11 |
| Monocytes | 4.929e-07 |
| NK cells | 2.235e-07 |
| Neutrophils | 6.595e-08 |
| Adaptive cells | 4.566e-06 |
| B cells | 0.0001 |
| T cells | 0.0001 |
| CD4+ T cells | 0.0002 |
| CD8+ T cells | 0.0459 |

**MIS-C vs. SAC**

| **Parameter** | **P-value** |
| --- | --- |
| Total cfDNA | 0.0098 |
| Innate cells | 0.0070 |
| Monocytes | 0.0340 |
| NK cells | 0.0340 |
| Neutrophils | 0.0151 |
| Adaptive cells | 0.6294 |
| B cells | 0.3270 |
| T cells | 0.9312 |
| CD4+ T cells | 0.9925 |
| CD8+ T cells | 0.6294 |

**SAC vs. CFR**

| **Parameter** | **P-value** |
| --- | --- |
| Total cfDNA | 0.0001 |
| Innate cells | 8.3111e-05 |
| Monocytes | 0.0013 |
| NK cells | 8.3111e-05 |
| Neutrophils | 0.0092 |
| Adaptive cells | 0.0406 |
| B cells | 0.0300 |
| T cells | 0.0440 |
| CD4+ T cells | 0.0440 |
| CD8+ T cells | 0.0511 |

**SUPPLEMENTARY FIGURES**

**Figure S1.** **T cells in MIS-C and SAC patients exhibit activated phenotype and increased migration out of peripheral blood.**


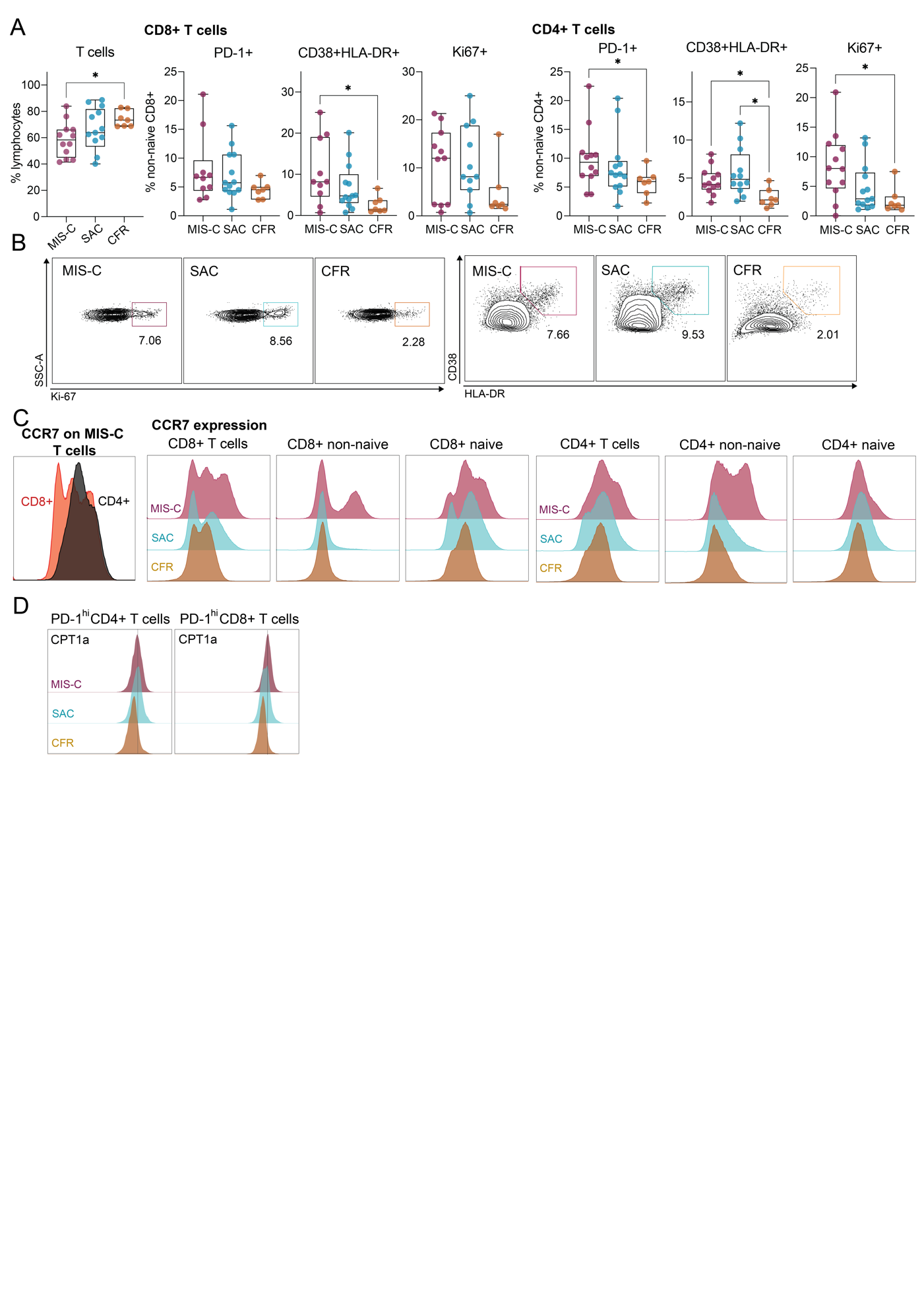


**A.** Frequency of T cells (%lymphocytes) in MIS-C, SAC and CFR groups. Compared to CFR individuals, a greater proportion of non-naïve CD4+ and CD8^+^ T cells in MIS-C and SAC patients express PD-1, HLA-DR and CD38, and Ki67. Wilcoxon test adjusted for multiple comparisons using Benjamini-Hochberg method, * p<0.05. **B.** Plots displaying Ki67^+^ and CD38 and HLA-DR double positive CD8^+^ T cells (similar trends were observed for CD4^+^ T cells). **C.** MFI plots of CCR7 expression on CD8^+^ and CD4^+^ T cells (total, non-naïve, and naïve subsets). **D.** Activated CD4^+^ and CD8^+^ T cells that express high levels of PD-1 co-express CPT1a.

**
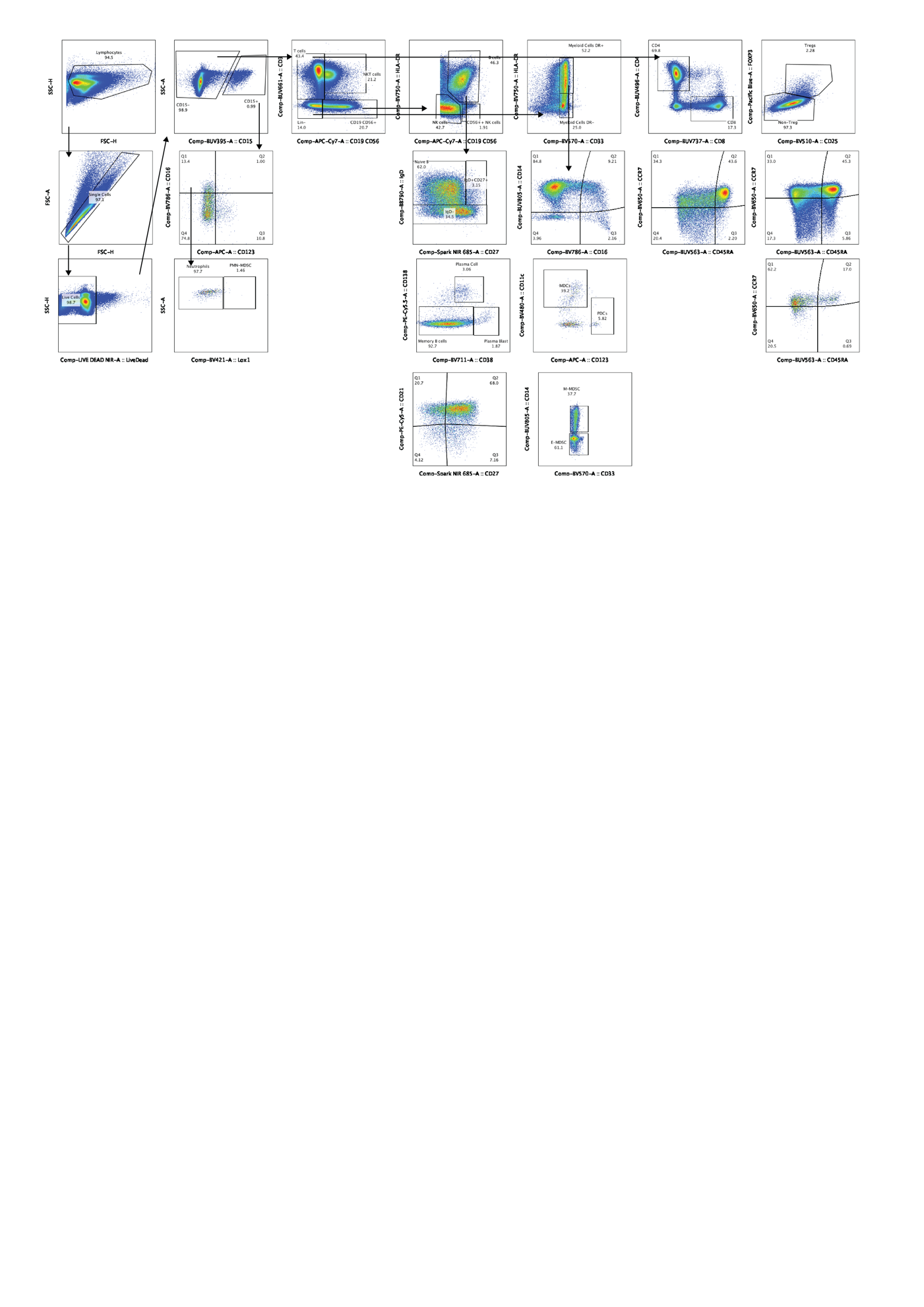
Figure S2. Representative flow cytometry manual gating strategy using the FlowJo software.**
